# First-Principles Investigation of the Adsorption Behaviors of CH_2_O on BN, AlN, GaN, InN, BP, and P Monolayers

**DOI:** 10.3390/ma12040676

**Published:** 2019-02-25

**Authors:** Chuang Feng, Hongbo Qin, Daoguo Yang, Guoqi Zhang

**Affiliations:** 1 School of Mechanical and Electronic Engineering, Guilin University of Electronic Technology, Guilin 541004, China; 1601201020@mails.guet.edu.cn (C.F.); d.g.yang@guet.edu.cn (D.Y.); g.q.zhang@tudelft.nl (G.Z.); 2 EEMCS Faculty, Delft University of Technology, 2628 Delft, The Netherlands

**Keywords:** monolayer materials, CH_2_O, first-principles calculation, gas sensor

## Abstract

CH_2_O is a common toxic gas molecule that can cause asthma and dermatitis in humans. In this study the adsorption behaviors of the CH_2_O adsorbed on the boron nitride (BN), aluminum nitride (AlN), gallium nitride (GaN), indium nitride (InN), boron phosphide (BP), and phosphorus (P) monolayers were investigated using the first-principles method, and potential materials that could be used for detecting CH_2_O were identified. The gas adsorption energies, charge transfers and electronic properties of the gas adsorption systems have been calculated to study the gas adsorption behaviors of CH_2_O on these single-layer materials. The electronic characteristics of these materials, except for the BP monolayer, were observed to change after CH_2_O adsorption. For CH_2_O on the BN, GaN, BP, and P surfaces, the gas adsorption behaviors were considered to follow a physical trend, whereas CH_2_O was chemically adsorbed on the AlN and InN monolayers. Given their large gas adsorption energies and high charge transfers, the AlN, GaN, and InN monolayers are potential materials for CH_2_O detection using the charge transfer mechanism.

## 1. Introduction

In the past years monolayer 2D materials have elicited increasing attention because of their superior thermal, mechanical, and optoelectronic properties [1,2]. Therefore, studies that focus on the applications of monolayer materials are attractive because these materials exhibit excellent performance in both gas sensors and optoelectronic devices [2,3]. With the development of the research, an increasing number of monolayer materials, such as boron nitride (BN) [4] and antimonene [5], have been predicted and synthesized. Within the monolayer materials, the family of nitrides has become a popular research material in optoelectronic and electronic applications because of their outstanding properties [4,6]. Two-dimensional (2D) BN, aluminum nitride (AlN), gallium nitride (GaN), and indium nitride (InN) materials exhibit a graphene-like honeycomb structure with band gaps of 4.47 [4], 2.9 [6], 2.16 [7], and 1.48 eV [8], respectively. Rao et al. successfully prepared graphene-like analogs of BN flakes, which can be applied in the preparation of composites [9]. It has been reported that an AlN monolayer can epitaxially grow on Ag (111) via plasma-assisted molecular beam deposition [10]. Graphene-like GaN monolayers exhibit high electronic mobility, indicating their excellent potential for application in nanoelectronics [11]. Moreover, first-principles calculations demonstrate that hexagonal InN monolayers exhibit excellent electronic properties [8], and InN nanowires can be experimentally prepared [12]. Furthermore, the boron phosphide (BP) monolayer is theoretically predicted to exhibit remarkable electronic properties [13], and a layered BP was prepared by Li et al. [14]. In addition, the blue phosphorus (P) monolayer was synthesized by Zhang et al. [15], and the monolayer P exhibited a remarkable potential for applications in nanodevices [16]. Ga, In, and P are critical raw materials for the EU while they are not critical for China [17], and the application of 2D materials can significantly reduce the amount of material necessary for the realization of a sensor.

Owing to their atomic thicknesses, large surface areas, and excellent electrical properties, monolayer 2D materials can become the new-generation sensitive materials of gas sensors [18]. In a previous study, the electronic characteristics of the monolayer materials were altered using some special adsorbed gas molecules [3]. Based on the investigation conducted by Zhou et al., P was ultrasensitive to NO, NO_2_, and NH_3_ gas molecules, indicating that the P monolayer is a potential material for the detection of these molecules [19]. Furthermore, antimonene was sensitive to NO, NH_3_, and SO_2_ gas molecules. Its electronic characteristics are altered after these gas molecules are adsorbed [3]. The investigation conducted by Li et al. indicated that monolayer MoS_2_ was suitable for detecting the O_2_, NO, and NO_2_ molecules [20]. Therefore, the application of monolayer materials in gas sensors is currently considered to be a popular research field.

Furthermore, various theoretical works and experiments have proved that BN, AlN, GaN, InN, BP, and P are potential sensitive materials that can be applied in gas sensors [19,21,22,23,24,25]. For example, ultrathin BN nanosheets can be used for detecting ethanol at high operating temperatures, and Lin et al. reported that the gas sensors showed a rapid response and recovery [21]. According to the investigation conducted by Wang et al., the AlN monolayer was suitable for detecting H_2_, CO, CO_2_, NO, and O_2_ via the charge transfer mechanism [22]. CH_2_O is a common toxic gas molecule that may lead to asthma and dermatitis in humans. Thus, detecting its presence and removing it from the environment is considered to be important. To the best of our knowledge, various traditional materials, such as ZnO [26], and NiO [27], have been considered for detecting CH_2_O; however, these materials have complex structures and work at high temperatures. Therefore, identifying new sensitive materials for detecting CH_2_O is useful. Several studies have exhibited that 2D materials may denote excellent gas detection performance [28,29]. In addition, investigations of the interactions between the CH_2_O molecule and the monolayer substrates BN, AlN, GaN, InN, BP, and P have been rare to date. In this study, the interactions between CH_2_O and the six monolayer substrates (i.e., BN, AlN, GaN, InN, BP, and P) were investigated using the first-principles calculation. This work can help researchers to study and predict the application of the six monolayer substrates in detecting CH_2_O.

## 2. Materials and Methods

All the calculations related to the interactions between CH_2_O and the monolayer substrates (i.e., BN, AlN, GaN, InN, BP, and P) were performed based on density functional theory (DFT) [30], using the Dmol^3^ package [31]. The generalized gradient approximation was performed using the Perdew–Burke–Ernzerhof (PBE) method [32]. For geometry optimizations, the convergence thresholds for displacement, energy gradient, and energy were considered to be 5 × 10^−3^ Å, 2 × 10^−3^ Ha/Å, and 1 × 10^−5^ Ha, respectively. While investigating the CH_2_O adsorption, the van der Waals interaction was considered to be critical. Hence, the method of Grimme [33] was employed while performing all the calculations. Furthermore, the Monkhorst–Pack mesh of 12 × 12 × 1 was selected for calculating the electronic structures. The gas adsorption system comprised a 4 × 4 supercell and one CH_2_O molecule inside, in which a vacuum of 25 Å was constructed.

To evaluate the gas adsorption strength between these monolayer materials and the gas molecules, the gas adsorption energy was defined as

*E*_a_ = *E*_(sub+molecule)_ − *E*_molecule_ − *E*_sub_ (1)

where *E*_(sub+molecule)_, *E*_molecule_, and *E*_sub_ denote the total energies of the adsorption system, CH_2_O, and substrate, respectively. Further, the charge density difference was calculated as

Δ*ρ* = *ρ*_(sub+molecule)_ − *ρ*_molecule_ − *ρ*_sub_ (2)

where *ρ*_(sub+molecule)_, *ρ*_molecule_, and *ρ*_sub_ denote the total charge densities of the adsorption system, CH_2_O, and substrate, respectively. Furthermore, the charge transfer (*Q*) was calculated using the Hirshfeld method, and the adsorption distance (*d*) was also calculated. The negative value of *Q* indicates that the CH_2_O molecule collects the electrons from the substrate, whereas *d* denotes the nearest distance between the substrate and the CH_2_O molecule.

## 3. Results and Discussion

**Figure 1:**
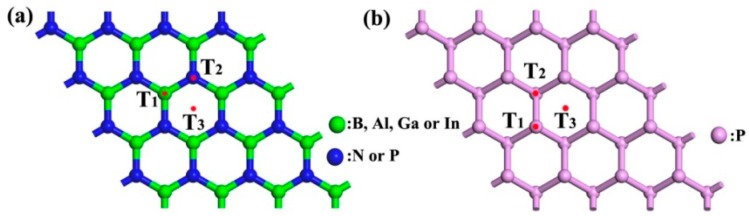
Three representative initial adsorption sites for (**a**) boron nitride (BN), (aluminum nitride) AlN, (gallium nitride) GaN, (indium nitride) InN, and boron phosphide (BP) and for (**b**) phosphorus (P).

**Figure 2:**
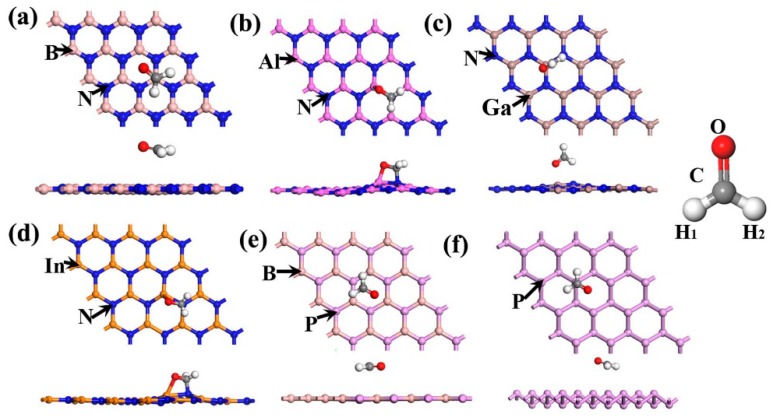
Most stable adsorption structures of CH_2_O on the surface of the (**a**) BN, (**b**) AlN, (**c**) GaN, (**d**) InN, (**e**) BP, and (**f**) P monolayers.

The structures of the BN, AlN, GaN, InN, BP, and P monolayers are 4 × 4 supercells, and the lattice parameters of the primary cells are 2.51, 3.13, 3.21, 3.63, 3.21, and 3.31 Å, respectively. The calculations of our structural constants were observed to be in good agreement with those of others [4,6,7,8,13,34]. While determining the most stable structures of CH_2_O on the BN, AlN, GaN, InN, and BP surfaces, three representative initial sites, including the top of X (herein after referred to as B, Al, Ga, In, and B) atoms (T_1_), top of N or P atoms (T_2_), and top of hexagon centers (T_3_) were considered, as plotted in Figure 1a. For the P monolayer three representative initial sites were calculated, as depicted in Figure 1b. After structural optimization, the most stable adsorption structures for CH_2_O on the surface of the BN, AlN, GaN, InN, BP, and P monolayers could be selected by choosing the lowest *E*_(sub+molecule)_ of the three initial adsorption positions T_1_–T_3_, as depicted in Figure 2. In the BN, AlN, GaN, and InN, monolayers, CH_2_O tended to be adsorbed on the top of B, Al, Ga, and In, respectively. After the gas adsorption of CH_2_O, the structures of AlN, GaN, and InN exhibited various deformation levels, whereas no obvious deformation was observed in the BN, BP, and P structures. In the subsequent discussion, all the results were calculated using these stable structures. 

The calculated values of *E*_a_, *Q*, and *d* for the most energetically stable adsorption structures are presented in Table 1. For CH_2_O on the surface of the BN, GaN, BP, and P monolayers, the values of *E*_a_ were −0.283, −0.456, −0.249, and −0.188 eV, respectively. Furthermore, the values of *E*_a_ for CH_2_O on the surface of pristine graphene was −0.162 eV [29], indicating that CH_2_O was easier to adsorb on the aforementioned materials than pristine graphene. The calculated values of *d* for BN, GaN, BP, and P were 2.990, 2.361, 3.328, and 3.230 Å, respectively, which were considerably larger than the bond length between the atom in CH_2_O and the substrate (i.e., *l*_H–N_ = 1.03 Å, *l*_O–Ga_ = 1.87 Å, *l*_O–B_ = 1.48 Å, and *l*_C–P_ = 1.38 Å, respectively) [35]. Thus, the adsorption of CH_2_O on these materials was considered to exhibit the trends of physical adsorption. The *E*_a_ values for CH_2_O on the AlN and InN surfaces were −1.044 and −1.046 eV, respectively, which were considerably larger than those of other materials. Meanwhile, the adsorption distances were 1.566 and 1.555 Å, respectively, which were in the bonding range (*l*_C–N_ = 1.46 Å), indicating that chemical adsorption may be observed in these two cases. 

**Figure 3:**
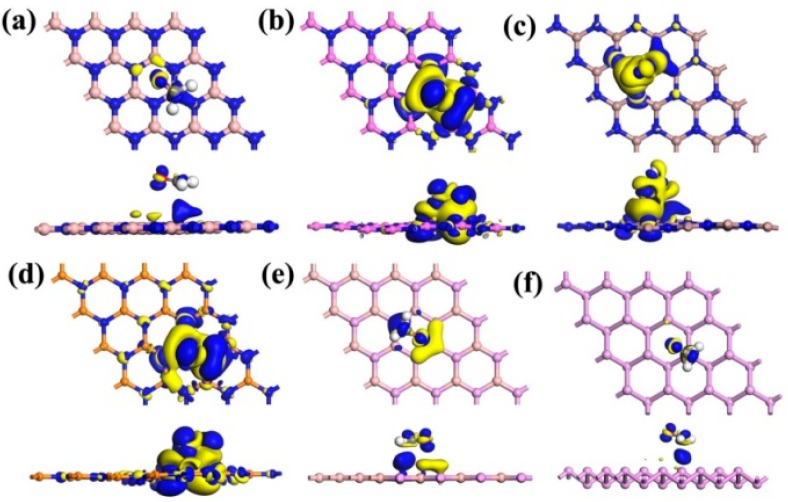
Charge density differences for the CH_2_O adsorbed on the surface of the (**a**) BN, (**b**) AlN, (**c**) GaN, (**d**) InN, (**e**) BP, and (**f**) P monolayers with an isosurface value of 0.003 e/Å^3^. The blue and yellow contours denote the increase and decrease of electrons, respectively.

Previous studies related to the (indium selenide) InSe and BP monolayers have demonstrated that the resistivity of the substrate can be altered by the adsorbed gas molecules using the charge transfer mechanism, implying that the value of *Q* plays an important role in the gas adsorption behavior [25,36]. The calculated values of *Q* for the CH_2_O adsorbed on the BN, AlN, GaN, InN, BP, and P monolayers were −0.019, −0.206, −0.107, −0.319, −0.065, and −0.067 e, respectively, revealing that the CH_2_O molecule gained electrons from these substrates. The *Q* values of the CH_2_O adsorbed on pristine graphene [29] and MoS_2_ [28] were −0.008 and −0.010 e, respectively, indicating that the charge transfers between CH_2_O and these materials were more noticeable than that to pristine MoS_2_ or graphene. To further investigate the charge transfers between CH_2_O and the substrates, the charge density differences were calculated, where the blue region represents an increase in the number of electrons, whereas the yellow region indicates electron reduction, as depicted in Figure 3. For CH_2_O on the surface of the AlN, GaN, and InN monolayers yellow regions were observed to be localized around the substrates, indicating that CH_2_O obtains electrons from the substrates. However, for CH_2_O on the surface of the BN, BP, and P monolayers, the blue or yellow regions were observed to be small, and the absolute values of *Q* were smaller than 0.07 e. These results indicate that the charge transfer in these cases was less evident when compared with that observed in case of the aforementioned materials (i.e., AlN, GaN, and InN). The charge transfers were reported to alter the number of charge carriers and resistance of the substrate [20]. Thus, the resistance of AlN, GaN, and InN may be noticeably altered after the adsorption of CH_2_O.

**Figure 4:**
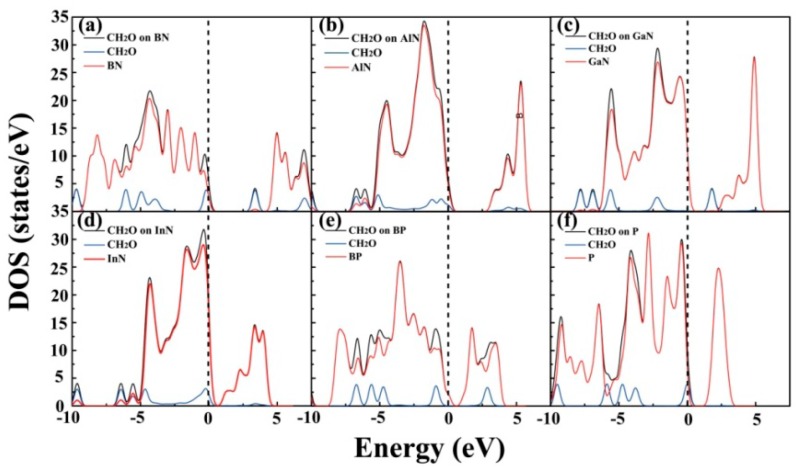
Density of states (DOSs) of CH_2_O on the surface of the (**a**) BN, (**b**) AlN, (**c**) GaN, (**d**) InN, (**e**) BP, and (**f**) P monolayers.

**Figure 5:**
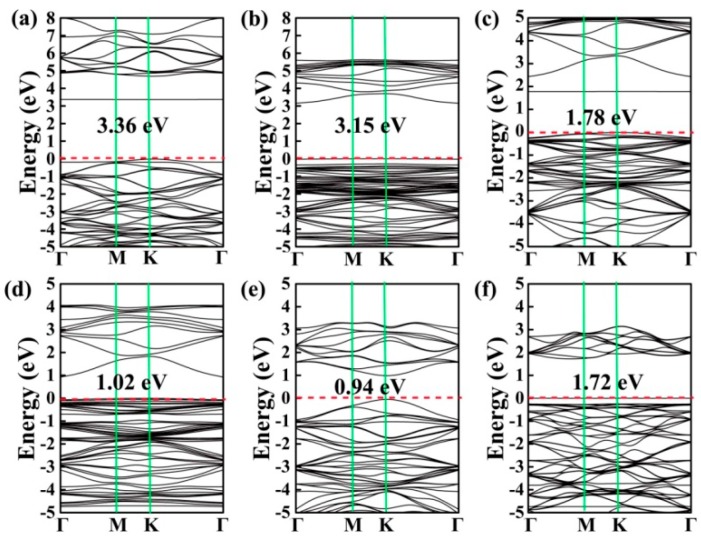
Band structures of CH_2_O on the surface of the (**a**) BN, (**b**) AlN, (**c**) GaN, (**d**) InN, (**e**) BP, and (**f**) P monolayers.

To perform an in-depth investigation of the adsorption of CH_2_O on the BN, AlN, GaN, InN, BP, and P monolayers, the density of states (DOSs) for the CH_2_O substrate systems were calculated, as depicted in Figure 4. Notably, for all the adsorption systems, the contribution of the electronic levels of CH_2_O was observed between −2.5 and 2.5 eV, which is around the Fermi level (*E*_f_). Note that the DOSs near *E*_f_ may exhibit a remarkable effect on the electronic characteristics of materials [3,22]. Thus, the existence of CH_2_O may have different degrees of influence on the electronic properties of these materials. To perform an in-depth investigation into the effects of CH_2_O on the electronic characteristics of the substrate, the band structures were also calculated, as shown in Figure 5. The band structure is an important parameter for determining the electrical properties of materials [24]. The band gaps of CH_2_O adsorbed on the BN, AlN, GaN, InN, BP, and P monolayers were 3.36, 3.15, 1.78, 1.02, 0.94, and 1.72 eV, respectively. The band gaps of pure substrates were as follows: *E*_g–BN_ = 4.67 eV; *E*_g–AlN_ = 3.43 eV; *E*_g–GaN_ = 2.45 eV; *E*_g–InN_ = 0.83 eV; *E*_g–BP_ = 0.94 eV; and *E*_g–P_ = 1.97 eV (refer to Figure S1 of “Supplementary Materials”). This result implies that the adsorption of CH_2_O had a noticeable effect on the electrical characteristics of these monolayer materials, except for BP. Because of the large band gap, BN is an insulator and not suitable for application in gas sensors. For CH_2_O on the surface of the P monolayer, the *E*_a_ and *Q* values were quite small even though CH_2_O influenced the electronic characteristics of the monolayer. This result indicates that P was not the most suitable material for detecting CH_2_O in our study. The AlN, GaN, and InN monolayers may have excellent potential for the detection of CH_2_O.

**Figure 6:**
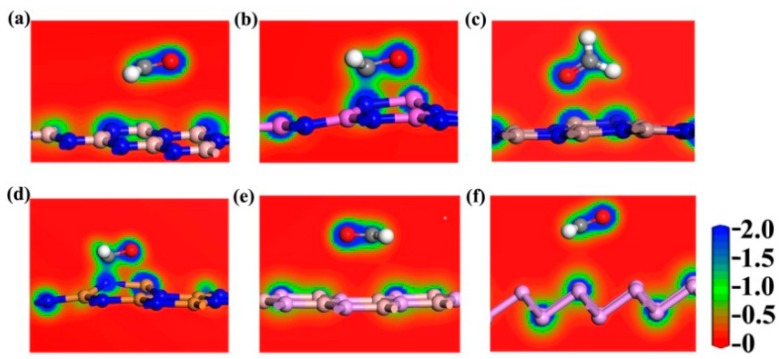
Slices of charge densities for the CH_2_O adsorbed on the (**a**) BN, (**b**) AlN, (**c**) GaN, (**d**) InN, (**e**) BP, and (**f**) P monolayers. The charge density ranges from 0 to 2 e/A^3^.

To obtain detailed confirmation about the type of gas adsorption behavior, the total electronic densities were also calculated, as depicted in Figure 6. The slices of electronic densities can help to determine the occurrence of a new chemical bonding. For instance, when CH_2_O is adsorbed on the BN monolayer, no obvious charge distribution was observed between the CH_2_O and BN atoms, and the adsorption distance was 2.99 Å, implying that the type of gas adsorption behavior was physical adsorption. Similarly, the gas adsorption behavior types of CH_2_O adsorbed on the GaN, BP, and P monolayers were also observed to follow the trend of physical adsorption. In the case of CH_2_O on the AlN and InN surfaces, the charge distribution between the CH_2_O and the substrates were apparent, considering the small adsorption distances, considerable adsorption energies, and large charge transfers, revealing the formation of new covalent bonds. As previously mentioned, the gas adsorption of CH_2_O was observed to noticeably influence the electrical properties of the AlN, InN and GaN monolayers. Given that the gas adsorption types of CH_2_O on the AlN and InN surfaces followed the trend of chemical adsorption, they exhibit an excellent potential for catalyzing CH_2_O or as disposable gas sensors for CH_2_O detection. For CH_2_O on the GaN surface, a chemical bond was observed between the gas molecule and the substrate. CH_2_O was therefore easily desorbed from the GaN monolayer after adsorption. Moreover, compared with In, the resources of Ga in China, USA, and the EU are considerable [17], indicating that GaN is suitable for use in gas sensors for CH_2_O detection.

## 4. Conclusions

In summary, the structure, charge transfers, and electronic characteristics of CH_2_O adsorbed on the BN, AlN, GaN, InN, BP, and P monolayers were investigated using first-principles calculations. For the adsorption of CH_2_O on the BN, GaN, BP, and P surfaces, the gas adsorption behavior followed the trends of physical adsorption. By assessing the band structures and DOSs of the gas adsorption systems, it was found that the electronic characteristics of these materials was evidently altered by the adsorption of the CH_2_O molecule, except for the BP monolayer. The GaN monolayer was considered to be the most suitable material for detecting CH_2_O in our study because of its appreciable charge transfer and moderate adsorption energy. The adsorption of CH_2_O on the surface of the AlN and InN monolayers was observed to follow the trends of chemical adsorption, with large charge transfers and considerable adsorption energies, revealing that AlN and InN have excellent potential for catalyzing CH_2_O or in disposable gas sensors for CH_2_O detection. 

## Figures and Tables

**Table 1:** *E*_a_, *Q*, and *d* of the gas adsorption systems of CH_2_O adsorbed on the BN, AlN, GaN, InN, BP, and P monolayers.

Substrate	Site	*E*_a_ (eV)	*Q* (e)	*d* (Å)
BN	T_1_	−0.283	−0.019	2.990 (H–N)
AlN	T_1_	−1.044	−0.206	1.566 (C–N)
GaN	T_1_	−0.456	−0.107	2.361 (O–Ga)
InN	T_1_	−1.046	−0.319	1.555 (C–N)
BP	T_3_	−0.249	−0.065	3.328 (O–B)
P	T_1_	−0.188	−0.067	3.230 (C–P)

## Supplementary Materials

The following are available online at https://www.mdpi.com/1996-1944/12/4/676/s1, Figure S1: Band structures of pristine (a) BN, (b) AlN, (c) GaN, (d) InN, (e) BP, and (f) P. monolayers.
