# Supplementary material for: First-Principles Investigation of the Adsorption Behaviors of CH2O on BN, AlN, GaN, InN, BP, and P Monolayers
Source: Materials (Basel). 2019 Feb 25;12(4):676. doi: 10.3390/ma12040676 (PMC6416566; doi:10.3390/ma12040676)
Supplement: Supplementary file 1 [file materials-12-00676-s001.pdf]

materials

Supplementary Materials

First-Principles Investigation of the Adsorption Behaviors of CH_2O on BN, AlN, GaN, InN, BP, and P Monolayers

Chuang Feng [1], Hongbo Qin [1,*], Daoguo Yang [1] and Guoqi Zhang [1,2]

[1] School of Mechanical and Electronic Engineering, Guilin University of Electronic Technology, Guilin, 541004, China; 1601201020@mails.guet.edu.cn (C.F.); d.g.yang@guet.edu.cn (D.Y.)

[2] EEMCS Faculty, Delft University of Technology, Delft 2628, The Netherlands; g.q.zhang@tudelft.nl (G.Z.)

* Correspondence: qinhb@guet.edu.cn; Tel.: +86-773-2290108;

Received: 29 January 2019; Accepted: 18 February 2019; Published: 25 February 2019

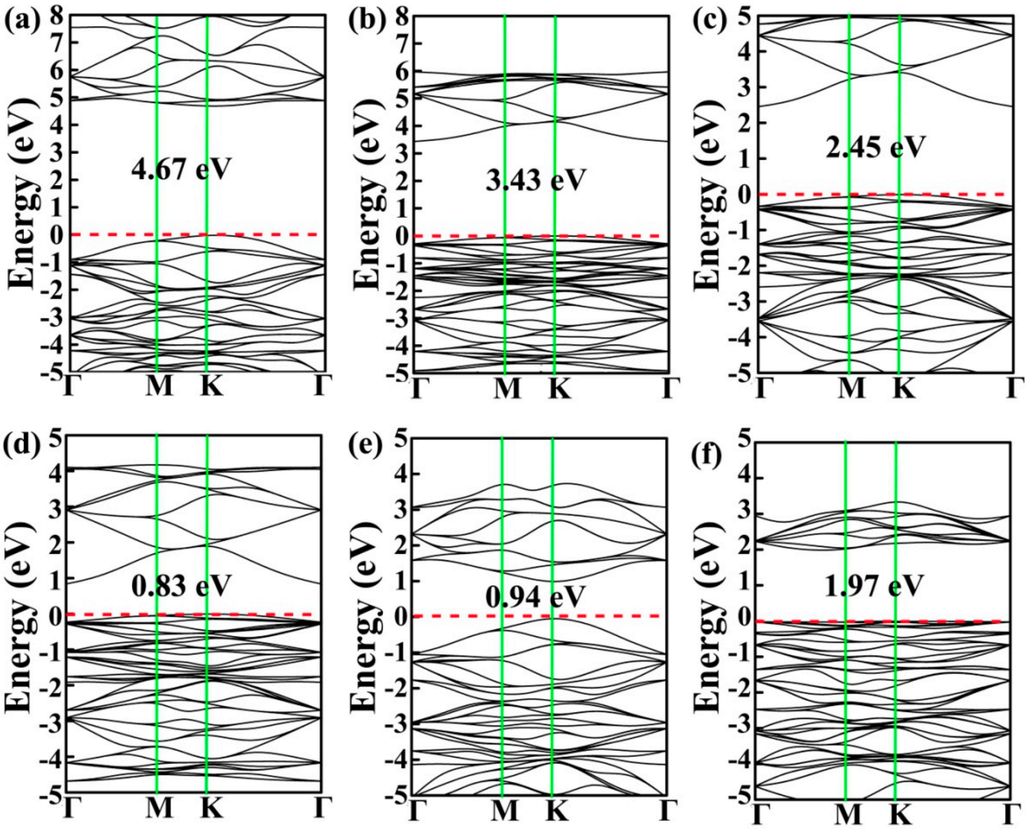

Figure. S1. Band structures of pristine (a) BN, (b) AlN, (c) GaN, (d) InN, (e) BP, and (f) P. monolayers.
